# Supporting New Community-Based Participatory Research Partnerships

**DOI:** 10.3390/ijerph16010044

**Published:** 2018-12-25

**Authors:** Nicolette I. Teufel-Shone, Anna L. Schwartz, Lisa J. Hardy, Hendrik D. de Heer, Heather J. Williamson, Dorothy J. Dunn, Kellen Polingyumptewa, Carmenlita Chief

**Affiliations:** 1 Center for Health Equity Research, College of Health and Human Services, Northern Arizona University, Flagstaff, AZ 86011, USA; Nicky.Teufel@nau.edu (N.I.T.-S.); Heather.Williamson@nau.edu (H.J.W.); 2 Department of Health Sciences, College of Health and Human Services, Northern Arizona University, Flagstaff, AZ 86011, USA; Dirk.deHeer@nau.edu (H.D.d.H.); kellenp@email.arizona.edu (K.P.); 3 School of Nursing, College of Health and Human Services, Northern Arizona University, Flagstaff, AZ 86011, USA; Anna.Schwartz@nau.edu (A.L.S.); Dorothy.Dunn@nau.edu (D.J.D.); 4 Department of Anthropology, College of Social and Behavioral Sciences, Northern Arizona University, Flagstaff, AZ 86011, USA; Lisa.Hardy@nau.edu; 5 Department of Occupational Therapy, College of Health and Human Services, Northern Arizona University, Flagstaff, AZ 86011, USA

**Keywords:** Community-Based Participatory Research, partnerships, American Indians, disability

## Abstract

Marginalized communities have a documented distrust of research grounded in negative portrayals in the academic literature. Yet, trusted partnerships, the foundation for Community-Based Participatory Research (CBPR), require time to build the capacity for joint decision-making, equitable involvement of academically trained and community investigators, and co-learning. Trust can be difficult to develop within the short time between a funding opportunity announcement and application submission. Resources to support community- and academic-based investigators’ time to discuss contexts, concerns, integration of expertise and locally acceptable research designs and data collection are limited. The National Institutes of Health (NIH) funded Center for American Indian Resilience and the Southwest Health Equity Research Collaborative have implemented an internal funding mechanism to support community and academic-based investigators’ travel cost and time to discuss complementary areas of interest and skills and to decide if moving forward with a partnership and a collaborative grant proposal would be beneficial to the community. The rationale and administration of this Community-Campus Partnership Support (CCPS) Program are described and four examples of supported efforts are provided. Centers and training programs frequently fund pilot grants to support junior investigators and/or exploratory research. This CCPS mechanism should be considered as precursor to pilot work, to stimulate partnership building without the pressure of an approaching grant application deadline.

## 1. Introduction

Community-Based Participatory Research (CBPR) integrates the expertise and perspective of both insiders and outsiders who contribute equally to a process of systematic inquiry and data collection, community capacity building and action [1,2,3,4,5]. CBPR requires time for individuals and institutions to develop trusted relationships to discuss community concerns and to integrate their collective capacity to develop solutions that use local assets and advocate change through program, system and policy development [6,7,8]. Time is needed without the looming requirement of making progress on research activities, for partners to gain familiarity with individual perspectives and values, work and communication styles, and long-term goals.

Recognizing the time and financial challenges faced by academic institutions, agencies, and communities interested in collaborating, the Center for American Indian Resilience (CAIR), a NIH National Institute on Minority Health and Health Disparities (NIH-NIMHD) funded P20 Exploratory Research Center of Excellence (2012–2017), developed a strategy to support new investigative partnerships, the Community-Campus Partnership Support (CCPS) Program. The success of this approach, assessed by the continuation of the partnerships beyond the CCPS funding and submission of co-developed grant proposals, led to the implementation of the support mechanism by the Southwest Health Equity Research Collaborative (SHERC) (2017–2022), a NIH-NIMHD U54 Research Center for Minority Serving Institutions (RCMI). Authors highlight four selected partnerships, and demonstrate how this approach has supported successful collaborations with research-cautious communities. These four selected as the community partners were willing to review or co-author a vignette of their experience for a peer-reviewed manuscript submission. This form of dissemination is often not valued in community settings and not covered under community partners’ scope of work. The intent of these descriptions is to illustrate the ways in which partners worked together to understand the expectations of their respective institutions and discuss skill integration. The intention is not to project community or university voice.

## 2. Building Partnerships and Trust

Trust can be difficult to build against the backdrop of negative perceptions about research in communities that have not been engaged as research partners, are marginalized, and/or have received few direct benefits from research findings [9,10,11,12,13]. Marginalized communities have more readily participated in CBPR than traditional research due to the emphasis on the equitable involvement of community members and local organizations throughout the research process [3,14,15,16].

Building trust and equitable partnerships in the competitive research environment is difficult. Federal, state, and private agencies that post Funding Opportunity Announcements require an application submission with 1–3 months of public dissemination of the opportunity. Three months is insufficient time for new partners to build meaningful relationships and have open dialogue about local concerns and potential value of research efforts. Investigators who approach agency and community leaders to discuss the benefits of partnership may appear anxious to develop a research plan and write a proposal. The term agency and community leaders is used broadly to include individuals who direct state, county, tribal and non-profit programs as well elected officials. The commonality is their position and ability to foster growth and positive change and their willingness to nurture resilience and identify assets in their clients and/or community. These leaders may be cautious to partner with a researcher who seems focused exclusively on the application and letters of support, overshadowing discussions about the health of the community. In contracts, partners who already have established relationships and researchers who have shaped their work around community-defined needs are more ready to develop proposals by demonstrating extended and productive relationships.

Partnerships begin in different ways. In some cases, researchers are involved with communities outside of their academic work and can build on those relationships. In other cases, leaders learn about positive experiences with a researcher or institution through inter-agency or community communication, and make contact with potential academic partners. On occasion, graduate students are invited to work in established CBPR partnerships and they in turn, are mentored into and can expand a community-university relationship.

The process for establishing new partnerships in the absence of pre-established links is less clear. Initial conversations can be awkward with investigators, agency, and community leaders trying to understand the others’ motivation, especially with regard to research goals as well as data collection and use. This uneasiness is compounded by the absence of resources and supports for potential partners to find time to meet face-to-face, especially if substantial travel and time are needed. Solutions often draw on the partners’ personal funds or discretionary funds and unfunded, sparse time is invested over the demands of academic and work environments.

## 3. Materials and Methods

The Community-Campus Partnership Support (CCPS) Program supports community, agency and academic partners to explore the role of resilience and identify local assets that contribute to health equity. CCPS funding ranged from $5000–$10,000/year for each partnership. Funds can be spent on travel and meeting support and/or community-based projects, but not on research activities. With satisfactory progress in their initial year, partnerships can reapply and receive support for a total of two years. In CAIR, the announcement was made and applications were accepted once/year. In SHERC, the announcement is posted and applicants accepted twice/year. This difference was driven by the amount of funds allocated to the respective centers and to community-engaged research.

Applications required a university and community agency partner to be named. In some cases, applicants knew of each other’s work and one reached out to the other to develop an application. In some cases, Dr. Teufel-Shone or Schwartz, lead developers of this mechanism and university based CPB researchers, knew both investigators and make introductions, suggesting they respond to the announcement. Partners that had a long-term relationship were not excluded from applying but were given lower priority in the final selection process, given the focus on stimulating new collaborations.

A minimal level of expertise is needed by both investigators to assure the partnership is grounded in familiarity with effective public health strategies. The community or agency partner is required to have worked for his/her employer for a minimum of 2 years. The academic partner must have an affiliation with a US based college or university as a graduate student, postdoctoral student, appointed personnel or faculty member and have a minimum of a 4-year undergraduate degree in a relevant field or a minimum of 5 years of relevant work experience. The opportunity announcement is posted on academic, county and tribal listservs, the CAIR and SHERC websites and was/is promoted by word of mouth by CAIR and SHERC personnel. In instances when community or agency partners contacted CAIR or SHERC for more information, CAIR or SHERC staff or faculty would suggest potential academic partners and in some cases, provide an email introduction.

The application requires CVs, resumes or bio sketches, and references for each partner, and a 2–5 page proposal providing a statement of problem, rationale for a partnership approach, proposed methods for building the partnership, expected outcome/deliverables, a timeline, budget and budget justification. Proposals are reviewed by 3 Center investigators using a standard scoring rubric and 3–5 partnerships are selected to receive CCPS funding each year.

Members of funded partnerships are required to attend a workshop (CAIR) that provides an introduction to peer-reviewed literature on resilience and positive deviance [17,18] or a webinar (SHERC) that provides an overview of CBPR principles and characteristics of successful community-university partnerships. Both the workshop and the webinar provide opportunities for past CCPS partnerships to share experiences and designate a workspace for new partners to delineate next steps to pursue their proposed plan. CAIR and SHERC provided resources and familiarized partnerships with external funding opportunities, strategies for collaborative grant writing, successful approaches to co-project administration, mixed data collection methods, tribal approval procedures for activities engaging university partners, and ethics related to research, evaluation and results dissemination.

Through the year, CAIR and SHERC mentored partners through regular conference calls, email discussions, or quarterly update reports to assure progress on projected milestones, problem-solve, and support teams to take advantage of opportunities for sharing experiences with community and scientific audiences. CAIR and SHERC supported career development in community and academic investigators to build a sustainable partnership.

At the end of the funded period, partners are expected to: (1) have gained experience in applying a collaborative framework in the design of an independent community-based project (e.g., community intervention or build sufficient institutional support to develop a grant application) and (2) adhere to a milestone schedule that yields tangible outcomes meaningful to both the community or agency and university. The latter expectation is linked to the critical observation that continued community-university partnership must demonstrate short-term benefits to research cautious community [19].

## 4. Results

From 2012–2016, 15 teams received CAIR partnership support. With the initiation of SHERC, eight teams were supported in the first two years (2016–2018). All funded partnerships completed their proposed work and the experience contributed to the professional growth of the community and university partners. Of the 23 funded, 13 of the partnerships are on-going. The work of three partnerships supported successful grant applications submitted by the partnering communities. Two partnerships shaped CBP research plans that were implemented as part of two junior investigators’ doctoral research. One partnership yielded a published, co-authored, peer-reviewed manuscript. The following four examples of community-university collaborative projects targeted key needs identified by communities and supported the initial step in integrating academic and practice-based expertise to address health equity. All featured partnerships are on-going. The first three were funded under CAIR in 2016 and the fourth under SHERC in 2018.

### 4.1. Improving Capacity for Healthier Homes

#### 4.1.1. Background

Project partners—a program director for healthy housing non-profit Red Feather Development Group, a tribal Community Health Representative (CHR) or lay health educators program and an associate professor of anthropology at Northern Arizona University (NAU) specializing in medical anthropology and community-engaged research—met through the CAIR Research Core Co-Director. Red Feather had strong community ties and brought in tribal leaders working in health and housing, along with staff from NAU’s Institute for Tribal Environmental Professionals (ITEP).

#### 4.1.2. Project Focus

At the outset of the partnership, project partners met in person in different locations over the first few months of the grant in order to identify common interests and complementary skills. They then developed the project to deliver training sessions to tribal Community Health Representatives (CHRs) on home health risk remediation, and to deliver health home kits for participating households. Poor indoor air quality, mold, and other home environment risks contribute to vast disparities among American Indians living on tribal lands. Project partners wanted to focus on reducing risks and supporting healthy homes.

#### 4.1.3. Approach

The project began with CHR participation in Red Feather training sessions which taught them about home environmental risk assessment through classroom-based instruction and home visits. These didactic sessions included chemistry-based information on indoor air quality and other home health risks as well as practical information on how to reduce risks for healthier homes. CHRs learned to use a standard home assessment instrument to identify risks such as drafts, mold, and other dangers through observation and discussion. Once classroom-based training was complete, the partners traveled together with CHRs to homes whose primary residents volunteered to receive a practice home assessment. CHRs walked around the inside and outside of houses using checklists to assess safety and environmental conditions of the structure. While providing access to their homes, occupants spoke to the partners present about their household and family relationships. During this part of the home assessment training, the anthropologist and an accompanying graduate student identified opportunities to expand the work beyond the structure of the home to the social and cultural dynamics of home health. They observed Red Feather employees walking past important cultural items in homes and going directly to holes in walls or stove dangers instead of stopping to ask about resilience and value attached to home. Drawing on this perspective, the medical anthropologist engaged processes at all future training sessions to talk with partners and collaborating CHRs about what would be appropriate and important to include on workbook materials for assessing homes. The household questionnaire now included asking residents about the meaning of their home, e.g., what would they want their grandchildren to know? What did they do in their homes that helped them to feel well? Another section was added to the end of the instrument to guide occupants to think about their available resources and ways to fix homes and reduce risks. The addition of these sections helped support cultural and locally appropriate aspects of wellness that people communicated were important for having a healthy home. Over the first six months of the project partners had frequent phone and in-person meetings to deliver trainings and also to talk with one another about different ways to approach these trainings sessions so they would be more community-engaged. The development of all materials, workbooks, and processes continued to deepen over the next few years of the project. Recently, Red Feather received funding for focusing on wood and coal stove safety. The anthropologist worked with project partners to identify the most pressing needs for safety in the partnering tribal community, and develop culturally and locally-relevant public service announcements based on that information to remind people to check their chimneys and remove fire hazards from around their stoves in the fall as temperatures drop in an attempt to reduce the risk of fires.

Throughout the project, partners shared differences in perspective and approach. Red Feather partners had to grapple with a shift in perspective that meant focusing on resilience and meaning beyond their primary goal of fixing homes. Tribal partners at first were unsure about the purpose of these new aspects of the trainings. The anthropologists were tasked with challenging Red Feather to think differently even within their already-established relationships of trust. In part, the ability to disagree and talk through these differences provided valuable experiences needed to build trust and expand each partner’s ability to work together toward solutions for improving wellness and home health. The time to have these conversations allowed the partners to have an ongoing conversation that continues today, four years later.

Drawing on their diverse perspectives, project partners developed and conducted several collaborative training sessions and workbooks. The team incorporated concepts of resilience and the social and cultural meaning of place in the initial training provided to CHRs. To supplement their activities, partners sought additional funding to expand training for CHRs and to collaborate with a tribal elder services program. The initial opportunity and subsequent projects shifted the work of each partner to integrate alternative perspectives in response to local needs. Community members gained new information about identifying and mediating household health risk in a locally relevant context. All partners built new relationships and developed effective collaboration skills. In their current work, four years later, project partners have integrated the strengths of each partnering entity. Tribal elder program coordinators, CHRs, and many community members who have enrolled in training sessions have the tools to focus on home health as a way to improve lives and reduce risks. They have learned structural information about home care, and incorporated cultural information about the meaning of home that elders want to pass on to family and community members. Red Feather has learned the value of including cultural information and resilience into assessments to support education and conversations about home repairs and risk remediation. Anthropologists expanded the team’s approach by sharing a community-based participatory process that consider the home residents’ perspective, and have increased their knowledge of indoor air quality and wood and coal stove burning risks and remediations specific to these communities.

The CAIR CCPS provided resources and time for partners to change the way they worked together and approached home health as part of a broad effort toward health equity on tribal lands. The result of this work has been increased trust among partners and improved strategies to promote home health. Partners are now fully engaged in ongoing projects in this area and have since added new partnerships and projects to expand the reach of this work.

### 4.2. American Indian Men’s Health: Next Steps

#### 4.2.1. Background

The partners in this project were a tribal member who worked as a community outreach coordinator with a tribal program responsible for provision of cancer support services and a NAU associate professor in Health Sciences who has experience on projects related to community health workers. Both partners had worked on the NIH National Cancer Institute (NCI) funded Partnership for Native American Cancer Prevention (NACP, U54CA143924/5), but on different Center cores. While they had interacted in Center meetings for several years, they had not collaborated directly prior to this project. During their initial interactions, both partners indicated they had a mutual interest in applied health promotion and recognized the need for cancer prevention at the tribal level. In addition, they shared an interest in focusing on groups that traditionally are less likely to seek care, such as American Indian (AI) men.

AI men suffer from an inordinate burden of illness and lack of utilization of health care services” [20]. Public health efforts could address these problems, presenting an opportunity to demonstrate resilience by restoring the strength of AI men and boys [21]. In 2012, prior to CAIR funding, a survey documented the tribe’s major health issues and demonstrated that men do not receive adequate provider direction about prevention and had insufficient knowledge about health and wellness screenings for the major health challenges (heart disease, cancer, and diabetes) [22,23]. Due to this shared interest, the collaboration was a natural fit to identify opportunities to bridge the gap between health providers and men in the community.

#### 4.2.2. Project Focus

The purpose of this project was to identify opportunities to work with the tribal Health Department to increase men’s use of health promotion services. CCPS funded project goals were met by talking with key stakeholders including community leaders, elders, staff of health providers and others, and summarizing recommendations as “next steps” needed to promote men’s health.

#### 4.2.3. Approach

The partners’ formative discussions to define the project topic and activities occurred in a relatively short period of time. Within two months from the initial discussion, the partners had developed the proposal and budget, defined their roles, and finalized plans for implementation. This process was expedited due to familiarity with each other’s work and overlap of interests. Following confirmation of funding, the team obtained Tribal Council approval and a memorandum of understanding (MOU) prior to initiating the Men’s Health project. The process of achieving the MOU took several months, in part due to different institutional and tribal perspectives and requirements for MOU language. The community partner served as a liaison with the tribal council, while the academic partner led navigation of the institutional processes. Following MOU approval, the community partner spoke with cancer survivors, community elders, Indian Health Services staff, cancer support services program staff, and public officials in the community. The discussions revealed concerns related to: (1) rates of cancer among men; (2) substance abuse; and (3) difficulty accessing screening programs because of transportation, work, and family commitments. Tribal members were aware of health and wellness programs on the reservation, but expressed confusion about the various programs and services because the Tribe has two health service providers, the Indian Health Services and the Tribal Department of Health and Human Services.

For tribal members, barriers to Indian Health Service and tribally-provided health care were transportation, lack of cultural empathy, and frequent medical staff turnover. For program administrators, barriers for improving or sustaining men’s health services centered on funding, as some programs relied on competitive grant awards. Tribal members preferred to use services and programs identified as being accessible and culturally-appropriate and -relevant (e.g., perceived as providing men with health information that benefit themselves and the health of their family).

This project illuminated local challenges and strengths impacting men’s use of health services; revealing previously unknown barriers. These outcomes are the result of working on a topic identified as important by the community and tribal leadership, as well as directly involving tribal partners as leaders on the project. Following the project, the academic partner’s research approach has shifted to rely on a collaborative leadership team that includes multiple PIs from both tribal and academic entities and a technical assistance relationship under tribal partner leadership. This approach has supported new funding for practical health promotion and policy-oriented projects that have strong support from partner communities and includes a focus on chronic conditions disproportionally affecting AI men. The community partner is continuing his education in public health while continuing to work for the center grant and a local federally recognized Urban Indian Health Center. The team continues to collaborate as key members of NACP contributing to both the Outreach, Training and Research Cores, and lending their skills to integrating activities across the different cores to a greater extent than before in the Center. The community partner has already been a part of several grant project submissions focused on promotion of AI men’s health issues, and both partners are actively exploring funding opportunities to continue to support and expand this work.

### 4.3. Promoting Resilience for Caregivers of Elder American Indians with Memory Loss

#### 4.3.1. Partnership Background

Project partners were introduced by a CAIR Research Core co-Director. The academic partner, an associate professor in NAU’s School of Nursing reached out to the Adopt-A-Native-Elder (ANE) by inquiring if she could provide an educational program to community members who care for elders with memory problems. ANE is a nonprofit organization that for over 30-years, has been providing assistance in the form of food, basic health care, clothing, fabric, and yarn to AI elders (ages 75- to over 100-years old), many of whom still strongly practice cultural and spiritual ways of living despite the historical, social, and political factors that have challenged their AI cultural identity [24]. Many elders live in locally constructed homes. In some cases, these homes are poorly maintained structures due to financial hardship and infrastructure challenges and maybe situated in remote locations on the reservations with no running water or electricity presenting some challenges for ongoing care and healthy living.

#### 4.3.2. Partnership Focus

To discuss how their skills and collaboration could benefits elders, the academic partner and the CEO of ANE started with regular phone calls to develop a relationship and discuss facilitating individual and group programs. The initial plan began on a weekly basis prior to the Food Runs by phone with the CEO of ANE. The purpose of the phone call meetings were to educate both the CEO about the program, memory loss, and healthy aging and the academic partner on how to be culturally sensitive with the AI elders. These phone call meetings took place over six weeks. The exchange of information was invaluable in preparing to deliver the educational program during the Food Runs. The purpose of the project that developed from the partnership was to explore the role of resilience in improving positive health outcomes in elders with memory loss and to provide education to interpreters, elders, and care givers.

#### 4.3.3. Approach

The academic partner attended six week-long ANE events known as Food Runs. Food Runs occur in the spring and fall and provide basic supplies such as fire wood, winter clothing, food items, and bottled water packed at the ANE Salt Lake City warehouses and transported to sites on the reservation. The day before each event, the ANE employees, volunteers, interpreters, and academic partner met to discuss logistics of a location of the teaching program and the overall layout of the Food Run location. Food Runs are usually held outdoors or an elder’s traditional dwelling. The academic partner was introduced to AI cultural health beliefs and the interpreters were introduced to non-AI understandings of normal cognitive aging and expectations as well as symptoms of cognitive decline. At the conclusion of each day, partners discussed what worked, what did not work and how they could make the program more effective the next day or at the next Food Run.

Outcomes of this CCPS collaboration included increased cultural understanding, awareness, and sharing for all partners. As an example of how this project expanded the understanding of partners, the non-AI academic partner learned that the word “dementia” did not resonate with cultural understandings of health and aging. At first, the non-AI partner used the term ‘memory loss’ but found it difficult to engage with the elders until an interpreter used the word ‘forgetful’. Discussing forgetfulness was quickly embraced by the elders as they shared their expectations, acceptance, and anticipation of becoming forgetful and felt their family would be there to help them. Once the non-AI partner understood that the elders expected their family to support them, the project focus shifted to providing support and education for caregivers who voiced frustration with repetitive questions, and forgetfulness of their elders. This program helped the ANE staff and volunteers to become more aware of the importance and hardships of the caregivers and not just the needs of the elders.

The collaborative project with ANE expanded the scope of the work of the academic partner to include AI people. She plans to continue the partnership and attend future ANE events with nursing students to offer psychoeducation support for caregivers. Nursing students will participate in the next phase of this project by assisting with educational programs and basic health screening (blood pressure and random blood glucose assessment) for caregivers and elders. ANE experienced the benefits of working with health care providers to improve its ability to prepare clients and their families to interface with non-AI medical providers and to bring resources to their clients. Screening services are often difficult for elders and their caregivers to access due to the rural geographical locations of the many communities on the reservation. This project will provide an opportunity for nursing students to deepen cultural humility and incorporate cultural competence in their nursing practice. Ideally, the collaboration will provide opportunities for AI nursing students to participate in programs that serve their communities.

### 4.4. Community Inclusion, Personal Development, and Health Equity among Youth with Intellectual and/or Developmental Disabilities

#### 4.4.1. Background

The primary partners on this project include an Assistant Professor (academic partner) in NAU’s Occupational Therapy program and a community leader (community partner) in employment support services for youth with intellectual and/or developmental disabilities (IDD). The academic partner has worked with individuals with IDD prior to returning to school for her doctoral degree in which she focused on health equity for adults with IDD. The community partner has worked for 37 years in the local area on efforts to support successful transition from school to adulthood, including finding gainful employment, for youth with IDD. IDD are diagnosed prior to age 22 years and involve the individual experiencing difficulty with daily practical and social skills due to physical and/or cognitive impairments [25]. Individuals with IDD experience health inequities and more efforts are needed to identify best practices for supporting their health equity throughout the lifespan.

The partners initially met during the academic partner’s year-long outreach to community members engaged in IDD support services in the region to inform this project’s development. The community partner invited the academic partner to join a Northern Arizona Community of Practice Transition Team (NACoPTT) group focused on supporting the success of youth with IDD in the region. The academic partner was fortunate to receive new investigator funding support from the American Occupational Therapy Foundation to complete a community-engaged photo story telling project with Native American youth with IDD focused on health and wellness [26]. Over the course of the next year, the community partner helped inform the scope and structure of the photo storytelling project, even co-hosting the project’s events at his place of work which was familiar to many young adults with IDD in the region. Throughout the original project, both partners stayed engaged on the NACoPTT efforts. The shared experience of working together on the coalition and on the year-long photo storytelling project led the partners to apply for CCPS funding through SHERC.

#### 4.4.2. Partnership Focus

Many adults with IDD experience loneliness, isolation, and limited opportunities for self-determined daily activities [27,28]. Through the photo-story telling project and discussions with the coalition, the partners identified that there are limited opportunities for youth with IDD to self-select meaningful participation in the community in order to explore their life goals and career interests. Service learning opportunities provide volunteer experiences to promote community engagement of youth with IDD and can assist the youth with IDD in self-determining their future career and educational pursuits.

#### 4.4.3. Approach

The partners are currently using the CCPS funding to engage in outreach efforts to identify potential community-sites for service learning for youth with IDD. The CCPS funding will allow for the hosting of open house events where members of the NACoPTT and youth with IDD can meet with potential service-learning sites to identify opportunities. Given the lack of knowledge regarding best practices for service-learning for the IDD community, the partners are also utilizing the funding to attend a national youth service learning conference to network with those leading service learning initiatives and discuss ways in which youth with IDD can be involved in those activities. The long-term goal will be to develop several service-learning opportunities in the region for youth with IDD. The partners hope to apply for additional funding to conduct research regarding the outcomes associated with service learning for youth with IDD.

## 5. Discussion

The CCPS funds efforts that use an interdisciplinary approach to build community and academic teams. The highlighted projects build upon the strengths of individuals and communities to identify local assets to increase access to and enhance the quality of health resources to address health equity. Each project required new partners to work through differences, expand their worldviews, and come to new places of understanding. For example, partners had time to identify local determinants of health outcomes including perceptions and use of medical services and ways the community talked about health issues, thus supporting the development of a common vision for change. This process supports Herbert and Best’s [29] observation that the challenges in health system innovation and change requires partnership to be grounded in shared values and the need for systems thinking.

The development of partnerships, not hindered by the negotiations required with research and data collection, allowed new connections to form. While refraining from data collection was uncomfortable for academic partners, the requirement supported an unfettered opportunity for partners to design an approach relevant to the community. The partners could consider innovative strategies together, a generative step that brought the new relationship to a meaningful step of joint purpose. This level of concordance can be lacking even in CBPR collaborations. Not collecting data has been framed as a limitation; however, in practice and hindsight this requirement is a key component to developing trusting relationships and building the foundation for ongoing work. Discussions about outcomes and goals between partners from different background and institutional settings are essential in the success of these highlighted projects and are facilitated directly by the structure of the CCPS Program. Each project focuses on a community-identified concern and develops a solution by combining the knowledge and local expertise of community leaders and the methodological skills of academic researchers.

The outcomes of these projects have positive direct influences on the individuals and communities involved, and have led to dissemination of information to public and private sector decision makers about health status, health behaviors, and health programs. They have also laid and continue to lay the groundwork for increasing successful partnerships between community and university-based investigators working together toward a common goal.

A limitation of the description of this funding mechanism is the absence of evaluation. Demonstrating the effectiveness of this funding mechanism could be assessed both short- and long-term. Short-term, the continued communication between partners demonstrates mutual recognition of each partner as a valued resource person with skills that could address a health concern. In these cases, partners may be working in-kind on community or leadership readiness to act by compiling or presenting local health statistics to program directors or other decision makers or on identifying an appropriate funding mechanism to support multi-year efforts. Long-term, the mechanism’s ability to support productive partnerships could be assessed through the outcome of successful collaborative funded projects and/or institutional relationships that support student internship opportunities or even recruitment with graduates working in communities.

Funding partnership formation provides the time and resources needed to build trusting relationships between partners free of the pressure of grant application deadlines. Once these relationships are built, partners can expand their work to include research where needed or desired by communities. The ongoing success of these partnerships demonstrates that providing non-research support to new partnerships is effective in establishing a foundation for ongoing collaborative work.

## 6. Conclusions

The partners engaged in these projects had resources and time to work collaboratively, and to experience the benefits of diverse perspectives and skill sets. CAIR and SHERC CCPS provides the space and time for partners to learn from one another and work together to design locally relevant approaches to community identified challenges. This small internal funding strategy supports the first step of CBPR, finding well-matched community-university research partners and eliminates the initial burden that can be felt by new partners to yield project outcomes while still building trust. This approach can be a precursor and funded initial step to pilot grant programs often offered by large funded Centers and Training grants to stimulate research.
